# Supplementary material for: Natural language processing augments comorbidity documentation in neurosurgical inpatient admissions
Source: PLoS One. 2024 May 9;19(5):e0303519. doi: 10.1371/journal.pone.0303519 (PMC11081267; doi:10.1371/journal.pone.0303519)
Supplement: Attachment — Submitted filename: Reponse to Reviewers.docx [file pone.0303519.s005.docx]

Rahul Sastry, MD

Department of Neurosurgery

Brown University

593 Eddy St, APC-6

Providence, RI 02903

rahul.sastry@gmail.com

March 24, 2024

Dear Dr. Kakulapati,

Thank you for providing a thoughtful review of our manuscript. We have attempted to clarify all of the points raised by the reviewers as follows:

*1. If the authors have adequately addressed your comments raised in a previous round of review and you feel that this manuscript is now acceptable for publication, you may indicate that here to bypass the “Comments to the Author” section, enter your conflict of interest statement in the “Confidential to Editor” section, and submit your "Accept" recommendation.*

*Reviewer #2: All comments have been addressed*

*Reviewer #3: All comments have been addressed*

*2. Is the manuscript technically sound, and do the data support the conclusions?

The manuscript must describe a technically sound piece of scientific research with data that supports the conclusions. Experiments must have been conducted rigorously, with appropriate controls, replication, and sample sizes. The conclusions must be drawn appropriately based on the data presented.*

*Reviewer #2: Yes*

*Reviewer #3: Yes*

*3. Has the statistical analysis been performed appropriately and rigorously?*

*Reviewer #2: Yes*

*Reviewer #3: Yes*

*4. Have the authors made all data underlying the findings in their manuscript fully available?

The* [*PLOS Data policy*](http://www.plosone.org/static/policies.action#sharing) *requires authors to make all data underlying the findings described in their manuscript fully available without restriction, with rare exception (please refer to the Data Availability Statement in the manuscript PDF file). The data should be provided as part of the manuscript or its supporting information, or deposited to a public repository. For example, in addition to summary statistics, the data points behind means, medians and variance measures should be available. If there are restrictions on publicly sharing data—e.g. participant privacy or use of data from a third party—those must be specified.*

*Reviewer #2: No*

*Reviewer #3: Yes*

*5. Is the manuscript presented in an intelligible fashion and written in standard English?

PLOS ONE does not copyedit accepted manuscripts, so the language in submitted articles must be clear, correct, and unambiguous. Any typographical or grammatical errors should be corrected at revision, so please note any specific errors here.*

*Reviewer #2: Yes*

*Reviewer #3: Yes*

*6. Review Comments to the Author

Please use the space provided to explain your answers to the questions above. You may also include additional comments for the author, including concerns about dual publication, research ethics, or publication ethics. (Please upload your review as an attachment if it exceeds 20,000 characters)*

*Reviewer #2: (No Response)*

*Reviewer #3: Clarity and Presentation:
The paper is well-structured and clearly presents the integration of natural language processing (NLP) for augmenting comorbidity documentation in neurosurgical inpatient admissions. The methodology and results are articulated effectively.

Originality and Contribution:
The paper addresses a significant issue in the healthcare domain, where comorbidity documentation is essential for patient care and treatment planning. The utilization of NLP to enhance this process is an innovative and valuable contribution.

Technical Soundness:
The technical aspects of implementing NLP for comorbidity documentation should be elaborated upon further. Explain the NLP techniques, tools, and algorithms used in detail. Provide insights into the data preprocessing, feature extraction, and model training procedures to ensure reproducibility.*

The methods section (page 4-8) has been greatly expanded to provide additional detail.  *Experimental Validation:
The paper mentions improved comorbidity documentation, but it would be beneficial to include quantitative results and metrics, such as precision, recall, and F1-score, to quantify the effectiveness of the NLP approach. A comprehensive evaluation would strengthen the paper's claims.*

Performance metrics have also been greatly expanded as suggested. This information is found in the results section.

*Data Privacy and Ethical Considerations:
Discuss the ethical considerations and data privacy safeguards implemented when handling patient data in a healthcare context. Ensure that all patient information is anonymized and de-identified to protect privacy.*

Further discussion of this point has been added to the discussion (page 17)  *Future Directions:
Conclude the paper with a discussion of potential future directions and applications of NLP in healthcare documentation beyond comorbidities. How can this technology be further extended to benefit patient care and streamline medical records?*

Further discussion of future directions has been added to the discussion on page 17.

*References:
Ensure that all references are up-to-date and accurately cited. Consider including recent works related to NLP applications in healthcare for a comprehensive literature review.*

We have ensured that the references are up to date.

*7. PLOS authors have the option to publish the peer review history of their article (*[*what does this mean?*](https://journals.plos.org/plosone/s/editorial-and-peer-review-process#loc-peer-review-history)*). If published, this will include your full peer review and any attached files.

If you choose “no”, your identity will remain anonymous but your review may still be made public.*

***Do you want your identity to be public for this peer review?*** *For information about this choice, including consent withdrawal, please see our* [*Privacy Policy*](https://www.plos.org/privacy-policy)*.*

*Reviewer #2: No*

*Reviewer #3: No*

Sincerely,

Aayush Setty

Rahul Sastry, MD

On behalf of all authors
