# Supplementary material for: Natural language processing augments comorbidity documentation in neurosurgical inpatient admissions
Source: PLoS One. 2024 May 9;19(5):e0303519. doi: 10.1371/journal.pone.0303519 (PMC11081267; doi:10.1371/journal.pone.0303519)
Supplement: Attachment — Submitted filename: Comments441.docx [file pone.0303519.s006.docx]

**Comments**

1. The related work section could be expanded to better contextualize the contributions and how they differ from other models.
2. Qualitative results showing example segmentation outputs could complement the quantitative results to better visualize the strengths/weaknesses.
3. The experimental setup and implementation details could be described more comprehensively to facilitate reproducibility.
4. Discussion on limitations, failure modes, and directions for future work is lacking.
5. Improve writing style, remove errors, grammatical mistakes.
